# Single cell RNAseq signatures refined with combiroc enhance identification of NK cells in blood and solid tissues

**DOI:** 10.1038/s41598-025-29876-5

**Published:** 2025-12-18

**Authors:** Ivan Ferrari, Saveria Mazzara, Andrea Gobbini, Noemi Di Marzo, Mariacristina Crosti, Sergio Abrignani, Renata Grifantini, Mauro Bombaci, Riccardo L. Rossi

**Affiliations:** 1 Istituto Nazionale Genetica Molecolare “Romeo ed Enrica Invernizzi”, Milan, Italy; 2 Department of Computing Sciences, Bocconi Institute for Data Science and Analytics (BIDSA), Bocconi University, Milan, Italy; 3 Artificial Intelligence & Systems Biology, IFOM-ETS The AIRC Institute of Molecular Oncology, Milan, Italy; 4 CheckmAb Srl, Milan, Italy; 5 Department of Pathophysiology and Transplantation, Università degli Studi di Milano, Milan, Italy; 6 Revelo Datalabs Srl, Milan, Italy; 7 Present Address: Research Computing & Data Science, IFOM-ETS The AIRC Institute of Molecular Oncology, Milan, Italy

**Keywords:** scRNA-seq, Gene signatures, Biomarkers, R package, Combinatorics, Phenotype overlap, Transcriptomics, Diagnostic markers, Bioinformatics

## Abstract

Cytotoxic CD8 T lymphocytes (CTLs) and natural killer (NK) cells share the common objective of controlling infections and detecting and removing tumor cells, albeit through distinct target recognition mechanisms. Although CTLs belong to the adaptive immune system and NK cells are innate lymphoid cells, they frequently exhibit considerable overlap in their molecular phenotypes. This overlap, as with many others in cell biology, poses challenges for distinguishing cells in the context of single cell transcriptomics. Building on a previous ROC-driven combinatorial approach, we developed a new computational framework for single-cell RNA-seq with the *combiroc* R package, and in this study we showed that it can identify non-canonical marker combinations for NK cells in Peripheral Blood Mononuclear Cell datasets. These combinatorial markers were in line with the Human Protein Atlas and we validated them through cytometry staining and functional assays. Markers selected with *combiroc* exhibit exceptional discriminatory power for identifying NK cells, both in blood and solid tumoral tissues. Besides this finding, we showed that our approach vastly optimizes standard differential expression signatures: it reduces dimensionality while improving interpretability and transferability to diagnostic applications, offering a practical solution for refining immune cell identities in complex transcriptomic datasets.

**Supplementary Information:**

The online version contains supplementary material available at 10.1038/s41598-025-29876-5.

## Introduction

Biomarkers derived from high-throughput omics techniques have revolutionized personalized treatment strategies and improved patient management^1–3^. However, the clinical viability of these biomarkers requires further investigation. Despite their transformative potential, research-focused omics methods often yield extensive gene signatures with limited discriminatory power, highlighting the urgency of identifying optimal markers from larger gene sets. We previously developed a diagnostic approach^4,5^ and applied it to research with diverse requirements^6,7^ but we faced challenges given by the nature of the data and the lack of control over the threshold assessment of experimental signals underlying such data. Moreover, gene signatures are made of tens, if not hundreds, of features characteristically expressed in a specific sample; single-cell RNA-seq refers to such signatures in thousands of units (i.e. cells) significantly scaling up the computational burden needed to process and find the best performing markers discriminating different cell cohorts. In this context, the most critical step in the selection of a subset of markers from larger signatures is the choice of the signal threshold above which a marker is considered to be positive, i.e. detectable above the baseline noise; this threshold is often relative to the nature of the assay, samples and the experimental conditions. Setting this detection threshold for markers can be challenging, or even impossible, when handling others’ data or poorly documented data, and is not well established for gene expression experiments at the single cell level. To overcome these limitations, either in the nature of the data or due to the computational burden we decided to develop a brand new R package, greatly improving the original method with new functions and broadening it to adhere to single-cell RNA-seq (scRNA-seq) standards^8^.

Besides data importing features from established scRNA-seq file format such as the Seurat object in the R single-cell workflow, combiroc automated the evaluation of detection signal distributions allowing automatic finding of the signal thresholds even for unknown datasets. We deployed the combiroc package on well-characterized scRNA-seq datasets and we asked if combinations of individual markers, smaller than the cognate gene signature they’re extracted from, were able to enhance the ability to discriminate between different cell type clusters, irrespective of the individual gene’s differential expression ranking in the original signature. Taking into account the known phenotypic overlap between NK cells and cytotoxic CD8 + T cells^9^, we identified new optimized gene signatures for NK cells, with comparable or even higher discriminatory capabilities than the larger parent signature in terms of sensitivity and specificity. To this aim we demonstrated that gene signatures based on strict differential expression ranking does not grant, per se, the best specificity and that characterization of cell clusters from scRNA-seq can be greatly improved by our refinement approach. The new *combiroc* package is fully documented and open sourced on the CRAN repository and has a continuously improving development version on GitHub: https://github.com/ingmbioinfo/combiroc.

## Results

### Automatic thresholding matches manual curation and improves downstream performance

**Fig. 1:**
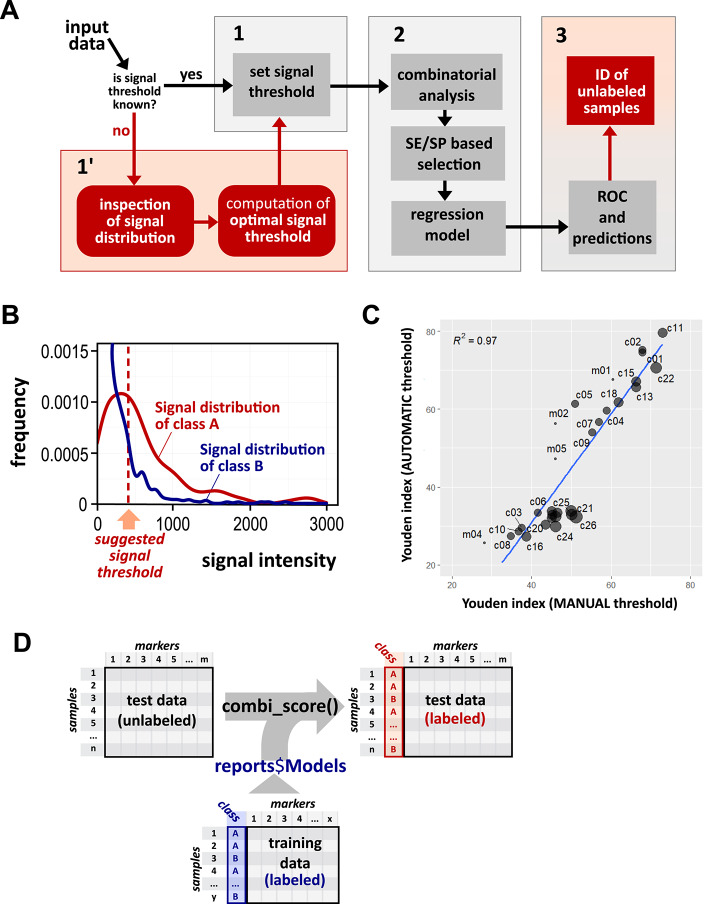
Combiroc automates signal threshold setting and creates models to annotate unlabeled samples. (**A**) Breakdown of combiroc’s workflow in phases (1, 1’, 2 and 3). Red boxes highlight new features specifically introduced by the R package: alternative phase 1’ for computation of signal threshold and part of phase 3 with labeling of unknown samples. (**B**) A typical output of the calculated optimal signal threshold (dashed line) on overlapping signal intensity distributions. (**C**) Correlation between Youden indexes for single AIH markers (m) and their combinations (c) computed after automatic and manual setting of signal thresholds. Dots’ size is proportional to the number of markers in each combination. Single markers from m1 to m5 are the genes IL4R, AQA598, RSPO3, CHAD and UNQ9419 respectively with their combinations as detailed in reference 4. (**D**) Samples of unlabeled data (left) can be associated with a class (right, red annotations), using regression models generated from labeled training data (blue annotations). “A” and “B” refer to the binary annotation of classes in the datasets.

In the process of identifying a subset of markers from a given signature the most critical step is the choice of a specific signal detection threshold which is strictly dependent on the nature of the assay. To address this, which is especially critical when working with datasets with unclear or not well-documented data, we introduced a new workflow to follow when the signal threshold is not known (Fig. 1A).

When a multi-marker signature for a pathological condition is available from a case-control study (or any two-class comparison), the distinction between case and control samples relies on a cutoff value that separates the detection signals of the two classes. Once marker signals for cases and controls are measured, an optimal threshold should positively select most samples belonging to the case class and negatively select most samples belonging to the control class. This threshold is usually set manually taking into account the distributions of the signals from the markers of the two classes. The new feature introduced by the combiroc package evaluates the signal distributions from both labeled sample classes, and automatically calculates a threshold value to be used in subsequent computations.

After pooling signal intensities from all single-markers a ROC curve is generated to discriminate cases from controls. Thresholds that meet minimal sensitivity (SE) and specificity (SP) requirements are considered. We then determined the Youden index which is calculated as (SE + SP – 1), so it ranges from 0 (useless) to 1 (perfect): this single score expresses how well each signal catches true positives avoiding false ones. This pooling serves only to rapidly identify a single global cutoff across markers. The resulting cutoff is then applied uniformly to binarize each marker as “expressed” or “not expressed”, while the actual combinations of markers are computed and evaluated only afterwards. The “suggested signal threshold” corresponds to the signal threshold value associated with the highest Youden index at which sensitivity (SE) and specificity (SP) are greater or equal to their user-set minimal values (min_SE and min_SP, both with default values at 0). Density distributions of signals from both classes are plotted and can be visually inspected, along with the suggested signal threshold value (Fig. 1B).

Using proteomic data from autoimmune hepatitis (AIH) patients^10^, previously analyzed in our work^4^ where the signal threshold for a 5-biomarkers signature was set manually, we aimed to verify how the signal threshold automatically suggested by the new combiroc procedure (resulting in 328.5) performed in downstream analyses compared to the manually defined threshold (equal to 450) used in the original work. We found that after the computation and ranking of all combinations with up to three markers, the top three combinations were the same (combinations #1, 2 and 11) with both thresholds setting methods; moreover, we showed that the Youden index computed for all the possible combinations with both thresholds (manual and automatic) are highly correlated, especially for those above Youden index 50 (Fig. 1C), thus confirming the validity of this approach. The AIH data used are available and preloaded as a demo dataset in the combiroc package, and code for the computations is detailed in the package’s main vignette (see Vignette 1 “Standard workflow” in Code Availability section).

A marker is as good as it is useful to annotate a specific cell identity. The manual annotation of cell identity is the standard procedure to determine cell populations but remains a limitation in most single-cell RNA-seq analysis^11^. Individual marker genes from signatures may lack specificity, often being differentially expressed across multiple cell clusters. Methods for the automatic or reference-based classification in single-cell RNA-seq exist, but established marker genes can be unreliable due to data sparsity^12^; moreover, rare cell types can lack unique markers for precise discrimination. We then extended combiroc’s usage to the single-cell RNA-seq analysis field as a signature refiner, in order to find the best few marker gene combinations from an already existing gene signature. To do so we decided to use the logistic regression models generated by combiroc using marker combinations from gene expression signatures of an annotated dataset to identify cells of the same class in unlabeled datasets with similar cell types (Fig. 1D). Before presenting its natural application as a gene signature refinement tool, we investigated whether combiroc could also serve as an effective cell classifier. To address this question, we benchmarked its classification performance against SingleR^13^, CellTypist^14^, and Azimuth^15^ using the F1 score as a comparative metric under a uniform preprocessing and label-harmonization workflow on three independent datasets containing similar cell types: cord blood mononuclear cells (CBMC) CITE-seq data from the “Seurat multimodal vignette”^16^, PBMC-3K^17^, and PBMC-Covid-19 single cell RNA-seq data^18^. Surprisingly, combiroc demonstrated competitive classification performance, achieving the highest F1 score in CBMC (0.881), ranking third in PMBC-Covid-19 (0.842), and the lowest in PBMC-3K (0.765) (Table S1). These results indicate that, although combiroc was not originally conceived as a classifier, it performs competitively—surpassing all other methods in one dataset out of three and being in line with the others tools for the remaining datasets. This highlights that no single classification approach performs optimally across all datasets, and that cell classification accuracy is inherently context-dependent, reflecting dataset-specific characteristics and annotation depth. Moreover, combiroc is overall competitive despite using only five features per class, whereas the other tools operate on thousands of genes, underscoring its efficiency and parsimony in capturing discriminative transcriptional profiles.

### Combinatorial refinement of NK cell signatures resolves overlap with CD8+ T cells

The individual specificity of top differentially expressed genes to their respective cell clusters remains a challenge, exemplified by the difficulty to distinguish NK (Natural Killer) cells from CD8+ T cells in many single-cell RNAseq datasets. This scenario provides a robust evaluation of combiroc’s efficacy in optimizing marker signatures. Starting from a NK-cell single-cell gene expression signature, our objective was to identify optimal gene combinations comprehensively describing NK-cell populations. The well-known phenotypic overlap between NK cells and cytotoxic CD8+ T cells posed a specific challenge due to the NKG7 gene’s high expression in CD8+ T cells, leading to misclassification. To address this challenge, we leveraged gene expression data from the multimodal peripheral blood mononuclear cells (PBMC) CITE-seq atlas, a widely recognized single cell RNA-seq public reference^19^ with its annotated cell clusters (Fig. S1A). We applied the standard differential expression Seurat protocol and we obtained the top 30 differentially expressed genes (DEGs) specific for the NK cells cluster (Table 1) confirming the phenotypic overlap. Notably, the top NK-specific genes in the obtained signature, including NKG7, were significantly detected in both NK and CD8+ T cells (Fig. S1B).

**Table 1:** Top 30 differentially expressed genes in NK. Differentially expressed genes obtained with standard differential expression Seurat v.4 protocol for NK cell clusters versus all other cell clusters. Numbers refers to ranking in the differential expression list.

1	GNLY	6	GZMA	11	CTSW	16	GZMH	21	GZMM	26	SYNE2
**2**	NKG7	**7**	KLRD1	**12**	CD247	**17**	IL2RB	**22**	MYOM2	**27**	CMC1
**3**	GZMB	**8**	CST7	**13**	CCL5	**18**	KLRB1	**23**	FCGR3A	**28**	EFHD2
**4**	PRF1	**9**	SPON2	**14**	CLIC3	**19**	TRDC	**24**	ARL4C	**29**	ADGRG1
**5**	FGFBP2	**10**	KLRF1	**15**	HOPX	**20**	CD7	**25**	ABHD17A	**30**	JAK1

**Fig. 2:**
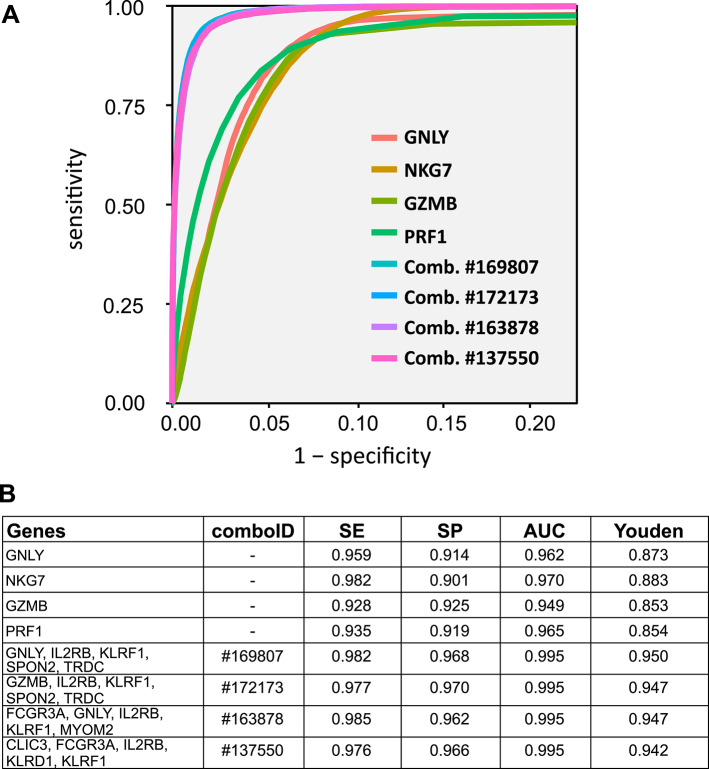
Performances of combiroc combinations compared to single top DE markers. (**A**) ROC curves for the top four individual NK markers and for the top four marker combinations. (**B**) Parameters for the markers (feature) depicted: SE, sensitivity; SP, specificity; AUC, area under the curve; Youden, Youden’s J index. All calculations and metrics are detailed in the package’s vignettes (referenced in Code availability section).

Using combiroc we explored all the possible combinations of up to five genes from the 30-genes signature, for a total of 174,436 combinations; we computed their SE and SP values and we ranked them based on their Youden index (Youden’s J = SE + SP -1)^20^. The four top performing combinations, each composed by five NK-cells marker genes, showed markedly higher SE and SP compared to individual top markers (Fig. 2; and reference of Vignette 2 in Code availability section).

### NK cells are correctly identified by combiroc in unlabeled single-cell datasets

To comprehensively evaluate the performance of combiroc-selected combinations, we conducted a thorough analysis on the three independent datasets containing similar cell types: CBMC^16^; PBMC-3K single cell RNA-seq data^17^, and PBMC-Covid19 multi omics data^18^ (Fig. S2). Across these datasets too the individual top four NK-specific genes (with an exception for PRF1 in PBMC-3K) failed to individually and effectively distinguish NK cells from CD8+ T cells, plasmacytoid dendritic cells (pDC), or gamma-delta T cells as shown by detectable expression levels in these cell clusters (Fig. S3). We used these datasets as an independent validation to evaluate the expression levels of the NK related marker combinations obtained with the training PBMC CITE-seq dataset. While we have been using the cell clusters annotation of the training dataset to differentiate NK cells from other cells, we instead removed cluster annotations from these three testing datasets, taking into account this information solely as ground-truth for evaluating combiroc’s performance.

**Fig. 3:**
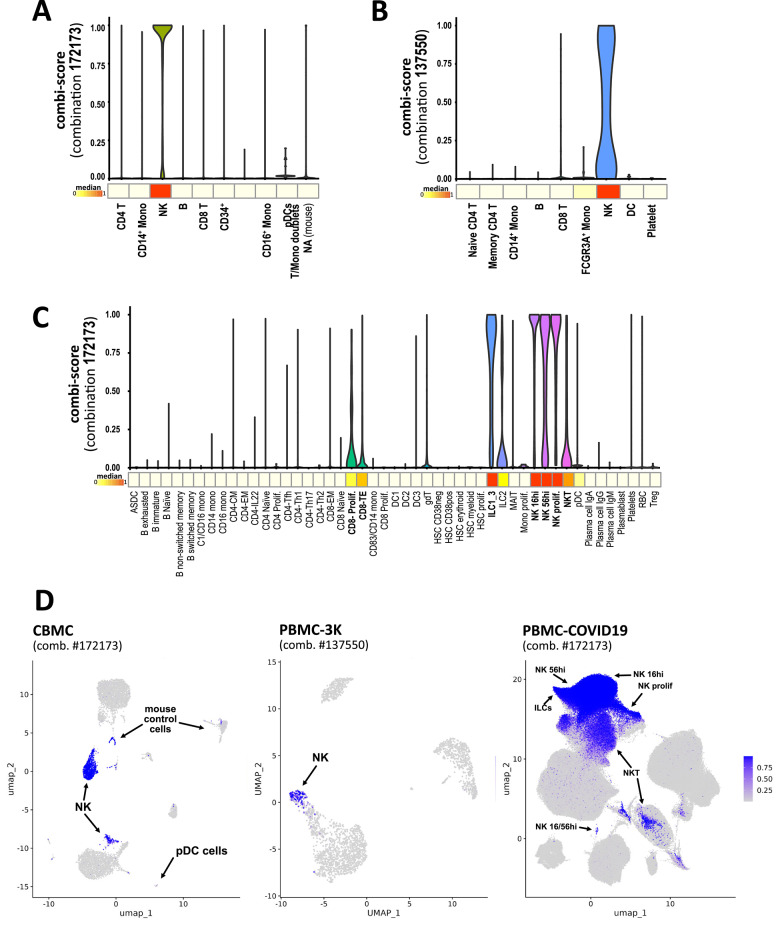
The combi-score from combiroc combinations correctly discriminates NK cells. (**A**), NK cells combi-score from combination #172173 seen as violin plot for each ground-truth cell cluster for CBMC dataset; (**B**) from combination #137550 for PBMC-3K dataset, and (**C**) from combination #172173 for PBMC-Covid19 dataset. The heatmap below x axis of violin plots shows the median value of combi-score for each cell cluster. (**D**), Combi-scores plotted on the UMAP feature plots of the same three datasets.

Based on the underlying regression model, we determined the predicted probability of belonging to NK cells. We named this probability “combi-score” since it can be determined using each selected marker combination with the package’s *combi_score()* function. We observed that the top combination (#172173, made of genes GZMB, IL2RB, KLRF1, SPON2, and TRDC) exhibited high specificity for cell clusters originally annotated with a “NK” label in the testing datasets, showing that combiroc positively predicted the dataset’s ground truth (Fig. 3A). Notably, pDCs , which in the same dataset highly express a NK marker such as GZMB, are totally excluded from the NK cohort by the combi-score; only a very small number of mouse cells included in the human CBMC dataset as a negative control were colored in the FeaturePlot (Fig. 3D), without relevance to the combi-score, as observed in the violin plots.

Similarly, in the PBMC-3K dataset, we computed combi-scores from combination #137550 (see Fig. 2B for genes), accounting for the absence of TRDC gene expression in this dataset, and it effectively identified the NK cells (Fig. 3B, D). In the PBMC-Covid19 dataset, combi-scores accurately identified various types of NK cells (CD16hi, CD56hi, proliferating), as well as Innate Lymphoid Cells (ILC), consistent with NK cells belonging to the ILC family^21^ (Fig. 3C, D). Across all three test datasets, combiroc-selected marker combinations consistently showed high accuracy in identifying the NK cell cluster and other specific cell types, highlighting the effectiveness of combiroc in precisely recognizing diverse cell populations.

### Gene combinations are more effective in discriminating cells than their longer parent signature

To further assess the efficacy of the combiroc-selected marker combinations, we employed an independent metric known as the “gene signature score”^22^, commonly applied to entire gene expression signatures obtained from differential expression analyses. We used this metric to compare the discriminatory power of the shorter gene combinations identified by combiroc with that of the longer gene expression signatures from which these combinations were derived. In order to do so, we calculated the gene-signature scores for both the entire 30-gene NK signature and the top 5-gene combiroc combinations in all datasets (#172173 in CBMC and PBMC-Covid19; #137550 in PBMC-3K).

**Fig. 4:**
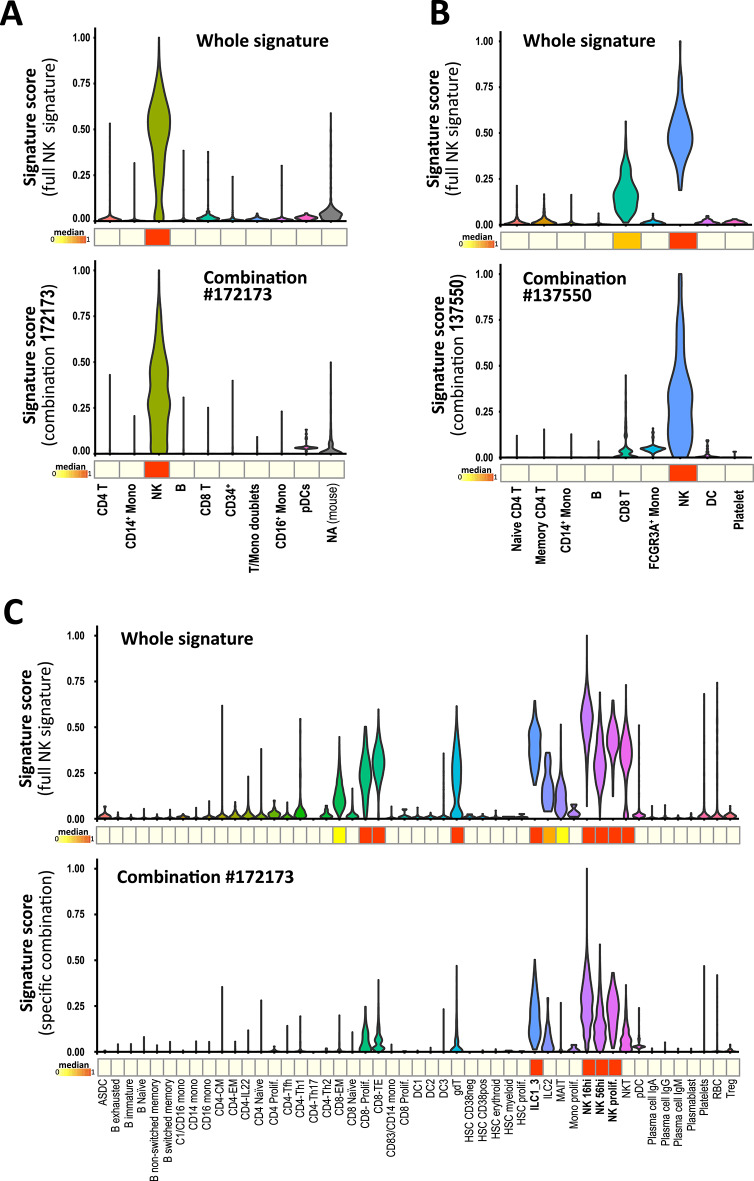
Selected combinations discriminate NK cells more clearly than parent signatures. Signature-scores determined from 30-genes NK cells whole signature (above) and from 5-genes combiroc driven combinations (below) computed across all cell clusters of (**A**) CBMC dataset, (**B**) PBMC-3K dataset and (**C**) PBMC-Covid19 dataset.

Notably, despite the sixfold reduction in the number of genes from 30 to 5, the scores derived from combiroc combinations were equally effective in identifying NK cells compared to those obtained from the complete signature, as shown for CBMC dataset (Fig. 4A). In PBMC-3K and PBMC-Covid19 datasets combiroc’s 5-genes combinations displayed even better discriminatory power with reduced noise, particularly in scenarios where the signals from CD8+ T cells and gamma-delta T cells were comparatively lower than those of NK cells (Fig. 4B-C). These findings highlight that combiroc’s marker combinations represent optimized gene signatures with comparable or even higher discriminatory capabilities than the larger parent gene expression signature.

### KLRD1 (CD94) and IL2RB (CD122) match conventional NK markers in specificity and cytotoxic readout

Upon inspecting the Human Protein Atlas (HPA)^23^ a repository of RNA expression and localization data, we found that the genes within the combiroc-selected combinations (Fig. 2B) were indeed associated with proteins annotated as enriched in NK cells, characterized by a very high Tau specificity score. Tau score is a numerical indicator of gene expression specificity across cells or tissues, ranging from 0 to 1, with 1 indicating exclusive expression in a single cell/tissue type. For the ten genes associated with the selected combinations, the median Tau score for cell type enrichment was 0.84. Half of these genes code for proteins localized to the cell membrane, while the other half for intracellular gene products (Table 2).

**Table 2:** Annotations from “Blood and Immune” cell types from the single cell data of human protein atlas (HPA, https://www.proteinatlas.org/*)* of the marker genes belonging to the selected combinations. Rightmost column reports the enriched cell types from single cell experiments in the HPA database with their Tau specificity scores.

Gene	Alias	Description	Localization	Enriched cell type (Tau specificity score)
IL2RB	CD122	Interleukin 2 receptor subunit beta	Membrane	*NK cells*,* T cells* (**0.89**)
KLRF1	CLEC5C	Killer Cell Lectin Like Receptor F1	Membrane	*NK cells* (**0.90**)
KLRD1	CD94	Killer Cell Lectin Like Receptor D1	Membrane	*NK cells* (**0.83**)
SPON2	DIL1	Spondin 2	Intracellular	*NK cells*,* dendritic cells* (**0.66**)
TRDC1	TCRD	T Cell Receptor Delta Constant	Membrane	*NK cells*,* T cells* (**0.93**)
GNLY	TLA519	Granulysin	Intracellular	*NK cells* (**0.84**)
FCGR3A	CD16	Fc Gamma Receptor IIIa	Membrane	*Monocytes*,* NK-cells*,* Macrophages*,* Hofbauer cells*,* Kupffer cells* (**0.84**)
MYOM2	TTNAP	Myomesin 2	Intracellular	*NK-cells* (**0.76**)
GZMB		Granzyme B	Intracellular	*Dendritic cells*,* NK-cells* (**0.89**)
CLIC3		Chloride Intracellular Channel 3	Intracellular	*Dendritic cells*,* NK-cells* (**0.70**)

**Fig. 5:**
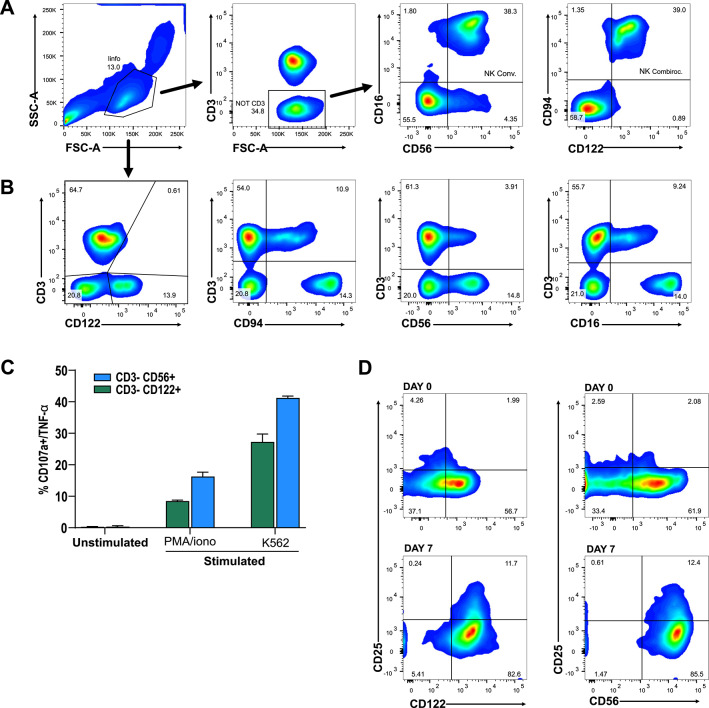
Combiroc selected NK proteins are specific to highly functional NK cells. (**A**) Gating strategy used to distinguish NK cell subsets. Live lymphocytes were identified based on side and forward scatter properties. CD3⁻ cells were subsequently analyzed for the expression of either CD56 and CD16, or CD94 and CD122. (**B**) Expression of the four NK-specific markers was assessed in comparison with CD3. (**C**), Fraction of CD3-/CD56+ and CD3-/CD122+ cells expressing CD107a after stimulation with PMA/ionomycin and with K562 cells. Percentages result from four independent experiments. (**D**) Expression of CD25 together with CD122 and CD56 at baseline (day 0) and after 7 days of IL-2 stimulation. Following IL-2 activation, NK cells upregulated CD25, while CD122 expression remained comparable to CD56, indicating that CD25 induction does not interfere with CD122 detection.

Through cytofluorimetric detection of membrane-localized proteins, we aimed to identify different cell types. We assessed the co-expression of selected membrane-associated markers, IL2RB (CD122), and KLRD1 (CD94), belonging to combiroc combinations, within the CD3-negative lymphocyte subpopulation. This was then compared to the co-expression of NCAM1 (CD56) and FCGR3A (CD16), the traditional markers used to sort NK cells^24^. The results revealed that these alternative markers from combiroc’s combination accounted for a substantial fraction (39%) of circulating NK cells, slightly more than the fraction (38.3%) obtained using common markers CD16+ and CD56dim/bright (Fig. 5A). Remarkably, even without gating out CD3 positive cells, the high expression of CD122 or CD94 exhibited an enrichment pattern similar to the one obtained with conventional markers (Fig. 5B).

To validate this finding, we conducted a cytotoxicity assessment of CD122-expressing cells through a degranulation assay, wherein the surface expression of CD107a is a marker of immune cell activation and cytotoxic degranulation^25^. After cell stimulation, anti-CD107a antibody was introduced, and after further incubation, the cells were stained for surface NK cell markers and intracellular tumor necrosis factor-α (TNF-α). Upon stimulation, CD107a expression markedly increased. While gating via CD122 resulted in a slightly lower fraction of CD107a⁺ cells compared to CD56, the functional responses – both degranulation and TNF-α production – were comparable. These data indicate that CD122 identifies functionally active NK cells and may provide a more specific, though not fully inclusive, perspective on NK cytotoxicity (Fig. 5C).

Interleukin 2 (IL-2) mediates its effect by binding to IL-2 receptors, namely IL2RA (CD25) and IL2RB (CD122). To assess whether IL-2 activation affects CD122 detection, peripheral blood mononuclear cells were isolated and NK cells were sorted using human CD56 magnetic beads. After 7 days of IL-2 activation, NK cells showed the expected up-regulation of CD25. Importantly, CD122 staining remained comparable when analyzed relative to CD56, indicating that IL-2-induced CD25 up-regulation does not interfere with the assessment of CD122 (Fig. 5D).

### The KLRD1/IL2RB marker pair (CD94/CD122) identifies NK cells in the primary tumor microenvironment

Although conventional NK cells represent the majority of CD56+ cells in most normal nonlymphoid tissues^26^, interpretation of studies regarding NK cells in solid tissues might be complicated by the use of markers that do not allow the distinction of *bona fide* NK cells from other cells present in solid tumor. We tested the selected combination of NK markers in tumor infiltrates derived from colorectal (CRC) and lung cancers.

**Fig. 6:**
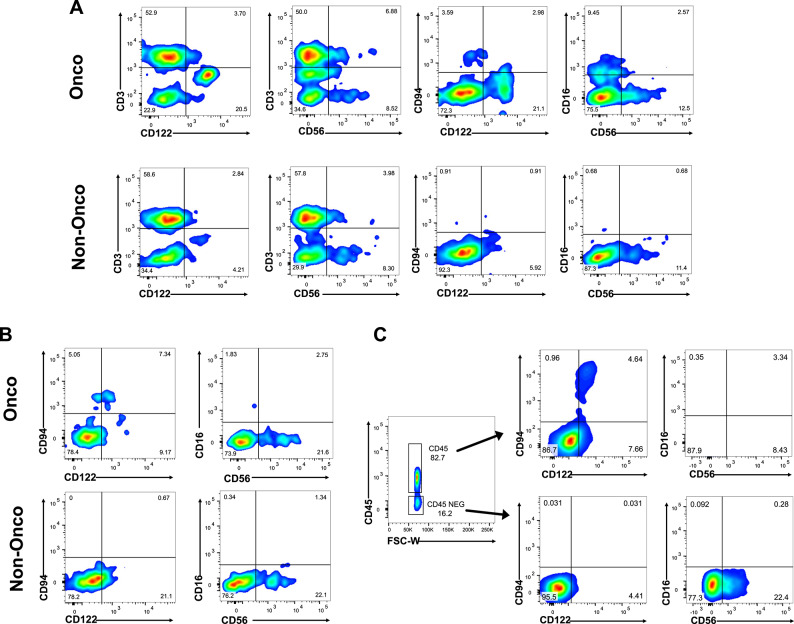
Identification of NK cells in solid tumors by CD94/CD122 expression. After tissue processing, tumor-infiltrating cells were stained with specific mAbs and analyzed by flow cytometry. (**A**) Analysis performed considering both CD3 expression and NK-specific markers. (**B**) Gating restricted to CD3⁻ cells to evaluate CD94/CD122 and CD56/CD16 expression in tumor and non-tumor regions. Data are from CRC with lung tissues showing similar patterns. (**C**) Gating strategy for sorting CD45+ cells (lymphocytes) and CD45- fractions after lung tumor processing, showing representative CD122/CD94 and CD16/CD56 staining.

After processing the CRC tissue, cells were stained with relevant antibodies and analyzed by cytofluorometry: the results revealed a comparable CD56 presence in both tumor and non-oncologic tissues even if CD56 expression is significantly higher than CD122, whereas CD122 exhibits wider differences in expression between non-oncologic and oncologic tissues (Fig. 6A). Moreover, when scrutinizing the same combinations in CD3-negative cells, the CD56/CD16 combination remains comparable between the tumor and non-tumor regions, while the CD122/CD94 combination was exclusively detected in the tumor region and not in the non-tumor area (Fig. 6B).

In lung cancer, the percentage of the CD56 marker can be overestimated because CD56 is also an epithelial marker^27,28^. To further assess the impact of CD56 on NK cell identification in lung tumors, we sorted tumor infiltrated cells into CD45- (not leukocyte cells) and CD45+ CD3- and with flow cytometry we analyzed the canonical CD16/CD56 combination, the CD122/CD94 (KLRD1/IL2RB) new marker pair or solely CD56. In the CD45+ cell fraction, the percentages of CD122 and CD56 overlapped (Fig. 6C, top panel). However, in the CD45-negative fraction, CD56 expression remains high at 22%, while CD122 is much lower at a fraction of 4%, indicating absence of noise from epithelial cells (Fig. 6C, bottom panel) underscoring the importance of meticulous marker selection and revealing by CD45 expression a distinct NK cell subpopulation in lung tumors. In conclusion, in these samples the identification of NK cells using KLRD1(CD122) and IL2RB(CD94) highlights the characteristics of NK cells more prominently expressed in the cancerous tissue compared to the healthy counterpart.

## Discussion

In this study, we addressed a longstanding limitation in the identification and annotation of NK cells in single cell transcriptomics developing a new R-based analytical framework with an improved version of our combinatorial procedure extracting optimized sub-signatures from differential expression gene signatures and confirming their efficacy through cytometry staining and functional analysis.

We demonstrated that the traditional top differentially expressed genes are not necessarily highly specific to their cell clusters but the combinations of markers selected with the combiroc framework described here were expressed on a similar or even larger proportion of functionally active target cells - in our specific case NK cells - compared to conventional markers. We believe that this approach holds significant utility in reevaluating existing traditional gene signatures, as well as new ones from omics experiments: by filtering out markers that genuinely matter, it enhances the robustness of cell identification driven by markers, effectively separating essential markers from the noise inherent in current high throughput methods.

While various statistical modeling strategies, such as threshold-based^29,30^, logistic regression^31,32^, and tree-based^33^ methods, and Support Vector Machines^34^, have been extensively employed to combine biomarkers, their practical application often remains within the domain of analytically skilled researchers. This limitation arises due to the complexities associated with extracting simplified, standardized, and interpretable results from these approaches^35–37^. With the increasing prevalence of transcriptome sequencing, obtaining a gene expression signature has become commonplace, and cell profiling resources^19^ are now an analytical commodity prone to be further used and optimized with methods that are able to seamlessly ingest single cell RNA-seq datasets. For this reason we added to the combiroc package the necessary interoperability with the widely used data objects from the Seurat package, which is the defacto standard for single cell analysis with R.

Acknowledging that a gene signature is context-dependent and always relative to the biological or experimental context in which it is determined, we emphasize the importance of refining signatures for enhanced performance. Noise from gene expression measurements and granularity of cellular annotations can compromise signature performance, particularly in more general contexts like healthy, non-diseased patients or common and multifactorial diseases. Despite this higher bias, one of the advantages of the approach shown in this work is a lower reliability on arbitrary assumptions in determining thresholds and cutoffs. First, the system has the ability to automatically select the most effective signal detection threshold, which is an additional element of difficulty when dealing with multiple markers since different positivity thresholds for each marker could be in place. Second, the numerosity of combinations (i.e. how many markers a good combination needs to be composed of) is a consequence of the statistical metrics used to rank them. The screening of combinations can and should be limited to those composed by no more than a few markers (we set the max_length to five in our search), since a lower number of well-chosen markers can be more efficient in cell labeling, as we showed using an independent metric such as the gene signature score (Fig. 4). While the choice of the maximum markers number allowed in a combination is indeed arbitrary, the reason to operate such a choice is twofold: first, it allows a decrease in the number of combinations to compute, making the analysis more manageable; second and more important, it fulfills the original aim of the package which is to search marker combinations significantly shorter than the original gene expression signature without compromising their discriminatory power, thus facilitating clinical applications of research results. We chose not to set a default for the markers’ number in the combination since the optimal maximum number can vary depending on the field of application and the experimental and/or clinical context. All of this is instrumental to the actual possibility to identify genes within a signature that are inherently more cell-specific, thus being crucial for accurate cell clusters annotation in single-cell transcriptomic experiments and for selecting the best-performing markers in biomedical or diagnostic settings, such as the tumor-infiltrating lymphocytes (TILs) or other critical domains like immune response profiling where precise cell identification plays a pivotal role in advancing our understanding and enhancing diagnostic precision.

This is exemplified by the standard tandem procedure of signature refinement, in which combiroc is used downstream of a classification step, often performed with other methods, operating on existing cell-specific gene signatures. The procedure works in principle for any type of cells across datasets. After Azimuth-driven classification on an additional independent dataset, the “10k PBMCs from a Healthy Donor (v3 chemistry)”, shortly PBMC-10K^38^, we identified a 30-marker gene signature overexpressed in CD8-T cells compared with all other cells in the dataset (Fig. S4A). From this 30-genes signature, we then used combiroc to determine the best combination of 5 markers (CD8A, CD8B, GZMH, LINC02446, TRAC) and observed that the cumulative expression of these genes shows a more specific pattern than either the whole signature or the top 5 differentially expressed genes (CD8B, CCL5, CD8A, CD3D, GZMH) (Fig, S4B-E). The amplitude of this effect is certainly cell-type dependent but can be observed across all cell types annotated in the dataset.

In conclusion, the combi-score derived from combiroc-selected marker combinations effectively discriminated among cell clusters and accurately identified cells in unlabeled datasets, utilizing a reduced subset of the original gene signature selected by combiroc. Our results demonstrated that the most specific genes for a cell subset are not necessarily the top differential expressed ones, or even the most established ones, and standard differential expression signatures from single cell RNA-seq can be further refined and optimized using combiroc, benefitting from the automatic signal threshold workflow characteristic of the package. The use of combiroc may empower researchers to confidently refine gene signatures, fostering a better identification of cell subsets, especially in the event of partially shared marker sets and phenotypic overlap. Finally, combiroc, when used downstream of gene signature detection in newly identified cell subsets, can streamline the manual annotation process by reducing the number of marker genes that needs to be considered, which is particularly useful for any diagnostic procedure or application.

## Materials and methods

### Extraction of the NK markers

The 30 genes differential expression signature from NK cells was determined from the Multimodal PBMC dataset from Hao et al. 2021 using the Seurat R package (version 4) and its standard protocol for differential expression with the function FindMarkers(SeuratObject, ident.1 = “NK”, ident.2 = NULL), then ordered by Fold Change and the top 30 hits were selected as NK signature.

### Model determination and marker combinations selection

To find the best combinations we used the *combi()* function. This function works on the training dataset by computing the marker combinations and counting their corresponding positive samples for each class (once thresholds are set). A sample, to be considered positive for a given combination, must have a value higher than a given signal threshold (signalthr) for at least a given number of markers composing that combination (combithr). As described in the combiroc’s vignette for the standard workflow (Suppl. Material 1 and GitHub at https://ingmbioinfo.github.io/combiroc/articles/combiroc_vignette_1.html), the argument signalthr of the *combi()* function should be set according to the guidelines and characteristics of the methodology used for the analysis or by an accurate inspection of signal intensity distribution. If specific guidelines or knowledge are missing, one should set the value signalthr as suggested by the distr$Density_plot feature. The screening of combinations was limited to those composed by no more than five markers (setting the max_length = 5 in the *combi()* function).

### Optimal signal threshold prediction

To predict the optimal signal threshold we used the *markers_distributions()* function, setting the argument *signalthr_prediction = TRUE*. In this way *distr$Density_plot* (see combiroc’s vignette for the standard workflow, Suppl. Material 1) will compute the threshold and show it besides the distribution of the signal intensity values for both classes; the threshold is computed as the median of the signal threshold values in *distr$Coord* at which SE and SP are greater or equal to their set minimal values (min_SE and min_SP). The optimal threshold is added to the “Density_plot” object as a dashed black line and a number, which is being used as signalthr value for *combi()* function.

### Training models on selected combination

Regression models on the selected combinations were trained using the function *roc_reports()*, which applies the Generalised Linear Model (*stats::glm()* with argument family = binomial) on each one. The equation used to compute the prediction is the following:

$$f(x)={\beta _0}+{\beta _1}{x_1}+{\beta _2}{x_2}+{\beta _3}{x_3}+ \cdots +{\beta _n}{x_n}$$

Where βn are the coefficients (being β0 the intercept) determined by the model and xn the variables (signal values associated to markers). The predicted probabilities have been calculated with the sigmoid function:

$$p(x)=\frac{1}{{1+{e^{ - f(x)}}}}$$

The performance of each model is internally evaluated in function of the cutoff (p(x) value above which an observation is positively classified) and an optimal cutoff is finally returned (cutoff at which occurs the least possible error of classification on the training dataset observations).

### Test datasets preprocessing

As independent validation to test the selected combination and models we used different single-cell RNA sequencing datasets to see if the obtained models were able to correctly identify NK cells, without having to rely on the original 30-genes NK-signature. The test datasets were loaded as detailed in the “Data Availability” section of this document. Both test datasets were harmonized following the same steps of the training dataset preparation (transposition, scaling values from 0 to 10, genes subsetting to the alphabetically ordered 30 marker genes and addition of ‘ID’ column) with the exception of the addition of ‘Class’ column which, obviously, was subsequently inferred in the end of the analysis by fitting the previously mentioned models.

### Combi-score

For each cell of the test dataset was computed a “combi-score” value (basically p(x)) using the standard *stats::predict()* method, specifying type=’response’. The combi score is, for each combination, the probability of the prediction of GLM fits on the scale of the response variable. This score was then used to assess the presence of cells classified as ‘NK’ in NK cells clusters: in this context, the combi-score is the probability of being a NK-cell given by a specific marker combination.

### Tests on unlabeled data

Test datasets were labeled by fitting each computed model with the *combi_score()* function following this logic:

- Cells with p(x) higher than the optimal cutoff are classified as “NK” (= 1).
- Cells with p(x) lower or equal to the optimal cutoff are classified as “Other” (= 0).
  $$C(x)=\left\{ {\begin{array}{*{20}{c}} {1\;\,p(x)>opt.\,cutoff} \\ {0\,\,p(x) \leqslant opt.\,cutoff\;} \end{array}} \right.$$

The performances of classification of each combination model were obtained by comparing the inferred labels with the originally annotated cluster labels.

### Gene signature score

For each cell of the test dataset was also computed a “gene signature score” to check the effect of using selected combinations on a different published score developed for whole genes signatures. The gene signature score is described in Della Chiara et al. 2021. It takes into account both the expression level and co-expression of genes within each single cell. Given a geneset, the increase of gene-signature-score is directly proportional to the number of expressed genes in the signature and to the sum of their level of expression. We reproduced the score computation with a custom R function described in signature_score.R script available in the GitHub combiroc package repository: https://github.com/ingmbioinfo/combiroc/blob/master/inst/external_code/signature_score.R.

### Signature refinement procedure

The PBMC-10K dataset was first annotated with RunAzimuth() function of the Azimuth R package version 0.5.0 using the first level of reference. The resulting object was then processed for a differential expression analysis with the standard single cell protocol (Seurat version 4); in particular, the Find Markers() function was used on cells classified as “CD8-T” and differential gene expression signatures were determined for this subset. The 30 top genes by fold change were selected and used as the combiroc input in the computation of all combinations made of up to five genes, first converting the Seurat object (seurat_to_combiroc() function), then using the combi() function to find and rank the combinations. The best 5-genes combinations were then ranked by Youden index and selected accordingly. The five genes belonging to the best combination were compared to the five top differentially expressed genes either by Youden index and by visual inspection on the whole cell population UMAP dotplot for cumulative expression.

### Collection of human biospecimens

CRC and lung cancers were obtained from pseudonymized patients recruited at Humanitas Research Hospital (Rozzano, Milan), European Institute of Oncology (Milan) in compliance with Ethic Committees (approvals IEO0849 June 27th 2018 and Studio 2868 April 21st 2021, respectively). Buffy coats were obtained from pseudonymized healthy donors from Centro Trasfusionale, IRCCS Ca’ Granda Ospedale Maggiore Policlinico, Milan in compliance with the local Ethical Committee (Study 3411_S_P December 13th 2023). All donors provided informed consent for the use of tissue samples, blood and blood derivatives for research use.

### Reagents, cell isolation, purification and tissue dissociation

Phorbol myristate acetate (PMA), ionomycin, monensin and Brefeldin A (BFA) were purchased from SIGMA-Aldrich. The K562 cell line was maintained in RPMI 1640 (Thermo Fisher Scientific) containing 10% FBS (GIBCO), 2 mM l-glutamine (GIBCO), 50 IU/ml penicillin (GIBCO).

Human peripheral blood mononuclear cells (PBMCs) were purified through density gradient centrifugation (Ficoll-Paque Plus; GE Healthcare). Solid tissues were extensively washed in PBS to remove cell debris and red blood cell aggregates. Subsequently, the samples were mechanically minced into small fragments using scissors. To minimize blood contamination, tissue specimens were rigorously rinsed after the initial fragmentation. Then, samples were enzymatically digested using a mixture containing DNAse (100 mg/ml), collagenase (1 mg/ml), and hyaluronidase (1 mg/ml) in RPMI 1640 supplemented with penicillin/streptomycin for 1.5 h at 37 °C. The resulting suspension was then filtered through a cell strainer and then washed by centrifugation in PBS to remove residual enzymes. Tissue-resident and blood mononuclear cells (MNCs) were isolated by Percoll (Sigma-Aldrich) density-gradient centrifugation to obtain the final tissue-cell suspensions. CD56^+^ NK cells were enriched with magnetic beads (CD56 MicroBeads, human, 130-050-401 Miltenyi) from PBMC using autoMACS Pro Separator (Miltenyi Biotec) and activated in vitro with IL2 at 2000U/ml of 7 day. All downstream analyses (CombiROC and cytometry panels) were performed on this highly purified NK population.

All research was carried out adhering to ethical, transparency and scientific fraud prevention policies of Istituto Nazionale Genetica Molecolare (INGM) and Fondazione Romeo ed Enrica Invernizzi.

### NK cell staining and flow cytometry analysis

For flow cytometry analysis, 2 × 10^5^ PBMC were stained and analyzed on FACSCANTO II (BD Bioscience). The following antibodies were used: CD56, CD3, CD122, CD94, CD16 as listed in table below. Briefly, cells were washed with MACS buffer (Miltenyi Biotech) and incubated with the antibody mix in MACS buffer at 37 °C for 20 min. Then the cells were washed and analyzed on an average of 10^5^ cells were acquired per sample, and data were analyzed using FlowJo software v.10.10 from BD Biosciences (https://flowjo.com/flowjo10/overview). For cell sorting of CD45 positive and negative fraction on lung tumor, cells were filtered using 50-µm filters (Filcons, Syntec International) and sorted on cell sorter BD FACSAria III SORP (BD Biosciences). Sorted cells were left in the incubator to recover for 2 h and then stained as described for NK cells.

### Cytotoxicity assay

For CD107 degranulation assay, PBMC were resuspended at 10^6^ cells/ml in complete RPMI 1640. Cells were then stimulated with MHC-devoid K562 cells, at an effector to target ratio of 10:1 in 96 wells plate at 200 µl final volume. Medium alone served as the negative control. Cells were stimulated with phorbol-12-myristate-13-acetate (PMA) (2.5 µg/ml) and ionomycin (0.5 µg/ml) (Sigma) as a positive control. Anti-CD107a antibody was added directly to the well at 1 µg. Cells were incubated for 1 h at 37 °C in 5% CO2 after which brefeldin A (Sigma) was added at a final concentration of 10 µg/mL as well as 6 µl of monensin (Golgi-Stop, BD Biosciences) at a final concentration of 6 µg/mL and incubated for an additional 5 h at 37 °C in 5% CO2. Cells were stained for surface NK cell markers for 20 min, were then fixed with 2% PFA for 15 min at room temperature, washed with PBS and permeabilized with 0.5% Saponin and stained for intracellular tumor necrosis factor-α (TNF-α) for an additional 30 min. After washing, cells were resuspended in MACS buffer and acquired on FACSCANTO II (BD) and analyzed using FlowJo software v.10.10 (BD Biosciences).

Antibodies used for this study.

**Table:** 

Marker	Clone	Catalog *n*°	Brand
FITC Mouse Anti-Human CD3	HIT3a	555,332	BD Pharmingen™
PE-Cy5 Mouse Anti-Human CD16	3G8	555,408	BD Pharmingen™
PE-Cy7 Mouse Anti-Human CD56	B159	560,916	BD Pharmingen™
APC Mouse Anti-Human CD94	HP-3D9	559,876	BD Pharmingen™
PE Mouse Anti-Human CD107a	H4A3	560,948	BD Pharmingen™
BV510 Mouse Anti-Human CD122	Mik-β3	563,093	BD Horizon™
BV421 Mouse Anti-Human TNF-α	MAb11	566,275	BD Horizon™
BUV395 Mouse Anti-Human CD45	HI30	563,791	BD Horizon™
APC-Cy7 Mouse Anti-Human IFN-γ	502,529	502,529	BioLegend

## Supplementary Information

Below is the link to the electronic supplementary material.

Supplementary Material 1

## Data Availability

All data used in this paper is available for download from public repositories and respective publication sources:- The multimodal PBMC (training) dataset is from Hao et al. 2021 and is available in GEO Omnibus database under the accession number GSE164378, and it can be directly downloaded as an h5 Seurat data (h5s) from the Fred Hutchinson & New York Genome Center Atlas page (https://atlas.fredhutch.org/nygc/multimodal-pbmc/)- The CBMC-CITE-seq (testing) dataset is from Linderman et al. 2022 and is available in GEO Omnibus database under the accession number GSE100866. It can also be directly loaded from the SeuratData package.- The PBMC-3K testing (dataset) is available from the Sequence Read Archive under accession number SRP073767, it can also be installed from the SeuratData library (https://github.com/satijalab/seurat-data) or downloaded from 10X Genomics repository (http://support.10xgenomics.com/single-cell/datasets). This dataset is licensed under the Creative Commons Attribution 4.0 International (CC BY 4.0 - https://creativecommons.org/licenses/by/4.0/) license.- The PBMC-Covid19 (testing) dataset is from Stephenson et al. 2021 and is available from Array Express under accession number E-MTAB-10026. It can also be retrieved from the Covid19 Cell Atlas (https://www.covid19cellatlas.org/index.patient.html) as “COVID-19 PBMC Ncl-Cambridge-UCL”.- The “10k PBMCs from a Healthy Donor (v3 chemistry)”, or PBMC-10K (used for tandem classification-refinement procedure) is among the official 10x Genomics Support dataset collection at: https://support.10xgenomics.com/single-cell-gene-expression/datasets/3.0.0/pbmc_10k_v3.

## Code availability

The combiroc package is available as a stable version from the Comprehensive R Archive Network (CRAN, https://cran.r-project.org/*)* and as an evolving development version from our GitHub repository (https://ingmbioinfo.github.io/combiroc/*).* The whole workflow with R code, datasets, precomputed objects, and commented results are available for review and reproducibility in the mentioned GitHub repository in the dedicated pages: ∙ Vignette 1: Standard workflow for the combiroc package: “Guide to CombiROC package - Standard workflow” at https://ingmbioinfo.github.io/combiroc/articles/combiroc_vignette_1.html. ∙ Vignette 2: Single cell sequencing complete workflow: “Signature refining tutorial - scRNA-seq Workflow” at https://ingmbioinfo.github.io/combiroc/articles/combiroc_vignette_2.html.
