# Supplementary material for: Single cell RNAseq signatures refined with combiroc enhance identification of NK cells in blood and solid tissues
Source: Sci Rep. 2025 Dec 18;16:358. doi: 10.1038/s41598-025-29876-5 (PMC12769544; doi:10.1038/s41598-025-29876-5)
Supplement: Supplementary file 1 — Supplementary Material 1 [file 41598_2025_29876_MOESM1_ESM.pdf]

Supplementary Figures for

Ferrari et al. “Single cell RNAseq signatures refined with combiroc enhance identification of NK cells in blood and solid tissues”.

Figure S1

A

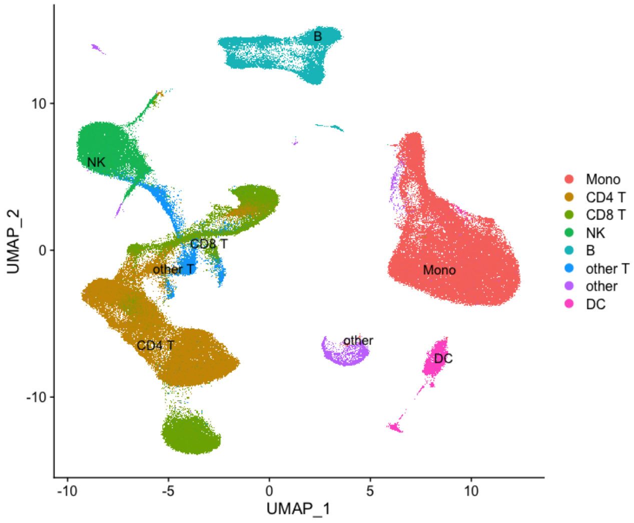

B

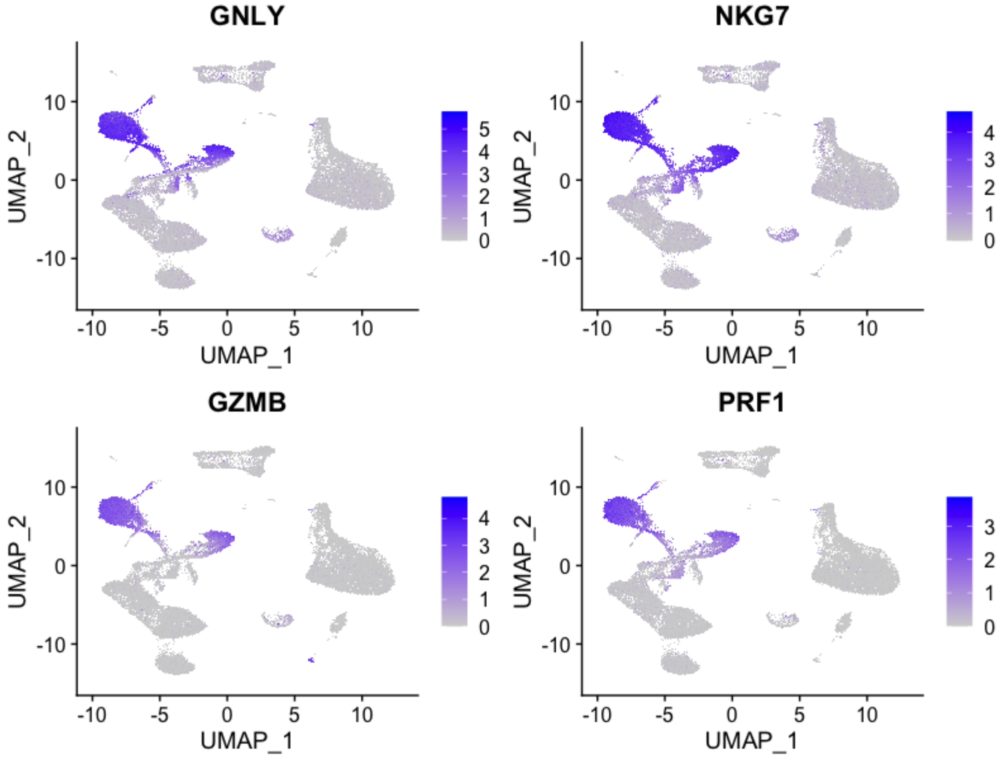

Fig. S1. Multimodal-PBMC CITE-seq atlas as training dataset.
(**A**) UMAP visualization of the annotated cell clusters. Cell clusters are annotated for eight different cell types according to gene expression and CITE-seq results in the original work. (**B**) Plots of the top four differentially NK cell markers belonging to the NK-gene expression signature showing that they are also highly expressed in other non-NK clusters (e.g. CD8 T).

Table S1

Dataset	Tool	TP	FN	FP	TN	SE	SP	Precision	Accuracy	Youden J	F1
CBMC	Azimuth	912	78	293	7334	0,921	0,962	0,757	0,957	**0,883**	**0,831**
	SingleR	911	254	294	7158	0,782	0,961	0,756	0,936	**0,743**	**0,769**
	Celltypist	880	325	56	7356	0,73	0,992	0,94	0,956	**0,723**	**0,822**
	Combiroc	1041	164	118	7294	0,864	0,984	0,898	0,967	**0,848**	**0,881**
PBMC-Covid19	Azimuth	14274	419	588	81758	0,971	0,993	0,96	0,99	**0,964**	**0,966**
	SingleR	14636	9824	226	72353	0,598	0,997	0,985	0,896	**0,595**	**0,744**
	Celltypist	14097	765	809	81368	0,949	0,99	0,946	0,984	**0,939**	**0,947**
	Combiroc	13270	1592	3390	78787	0,893	0,959	0,797	0,949	**0,852**	**0,842**
PBMC-3K	Azimuth	148	8	7	2475	0,949	0,997	0,955	0,994	**0,946**	**0,952**
	SingleR	155	90	0	2393	0,633	1	1	0,966	**0,633**	**0,775**
	Celltypist	111	43	5	2479	0,721	0,998	0,957	0,982	**0,719**	**0,822**
	Combiroc	109	46	21	2462	0,703	0,992	0,838	0,975	**0,695**	**0,765**

Table S1. Benchmarking against Azimuth, SingleR and CellTypist. TP: true positive, FN: false negative, FP: false positive, TN: true negative, SE: sensitivity, SP: specificity.

Figure S2

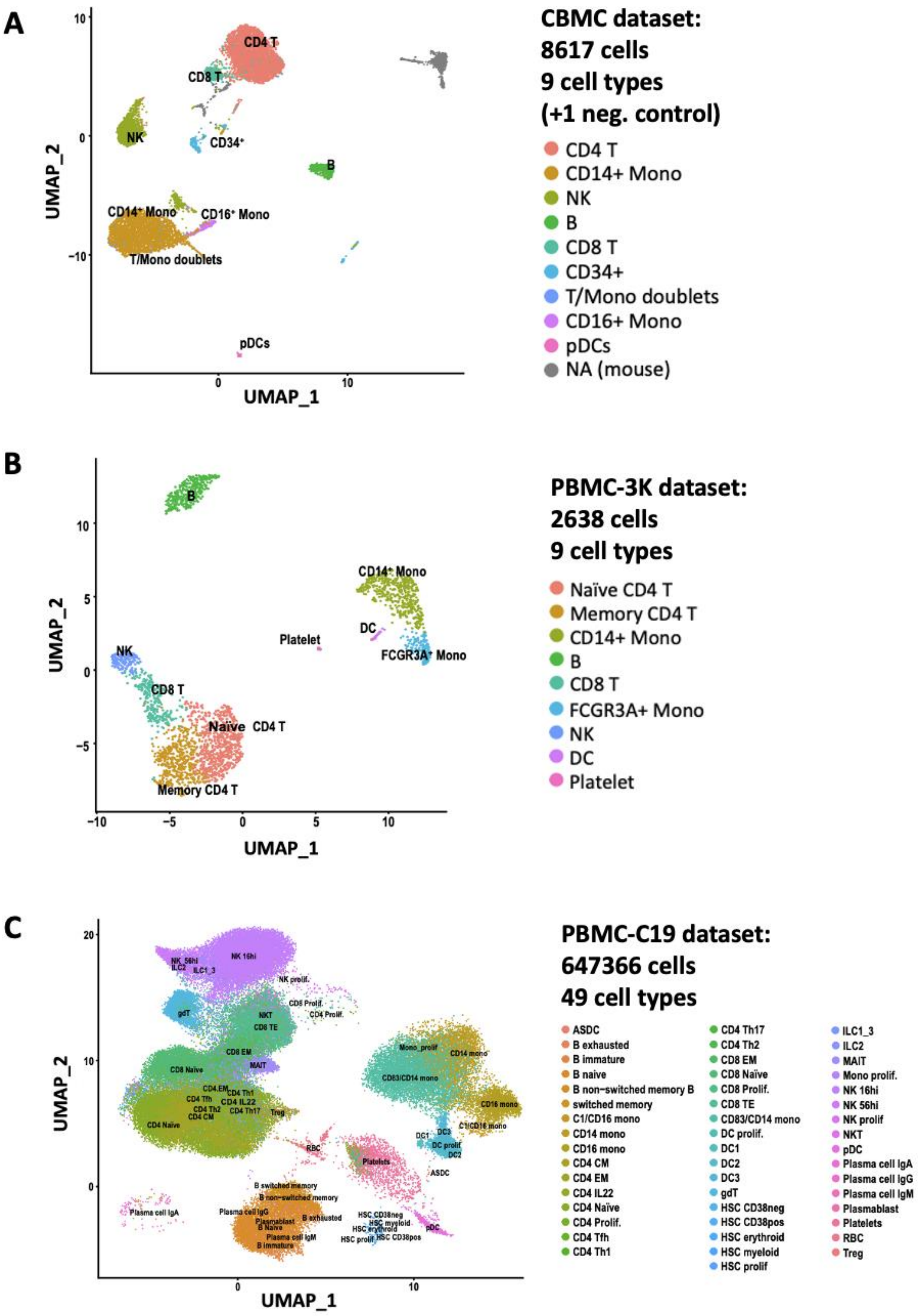

Fig. S2. Cell cluster annotation of testing datasets used (**A**), UMAP visualization of CBMC dataset, gray cluster labeled with “NA” is a negative control made of murine cells; (**B**) PBMC-3K dataset and (**C**) PBMC-Covid19 dataset.

Figure S3

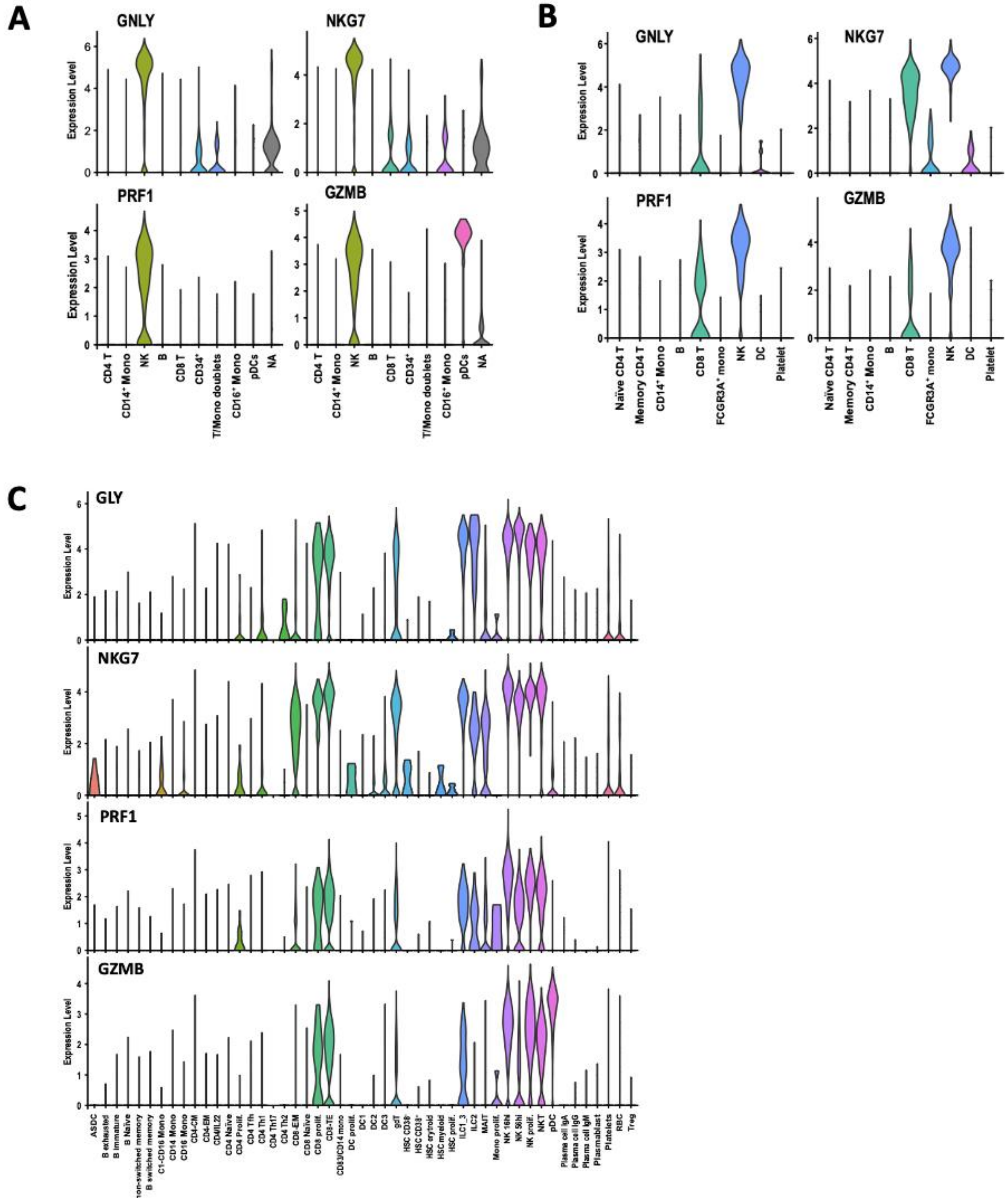

Fig. S3. and violin plots for cluster specific expression of the top four individual gene markers. (**A**), Violin plots for cluster specific expression of the top four individual NK gene markers in CBMC dataset, (**B**) in PBMC-3K dataset, and (**C**) in PBMC-Covid19 dataset.

Figure S4

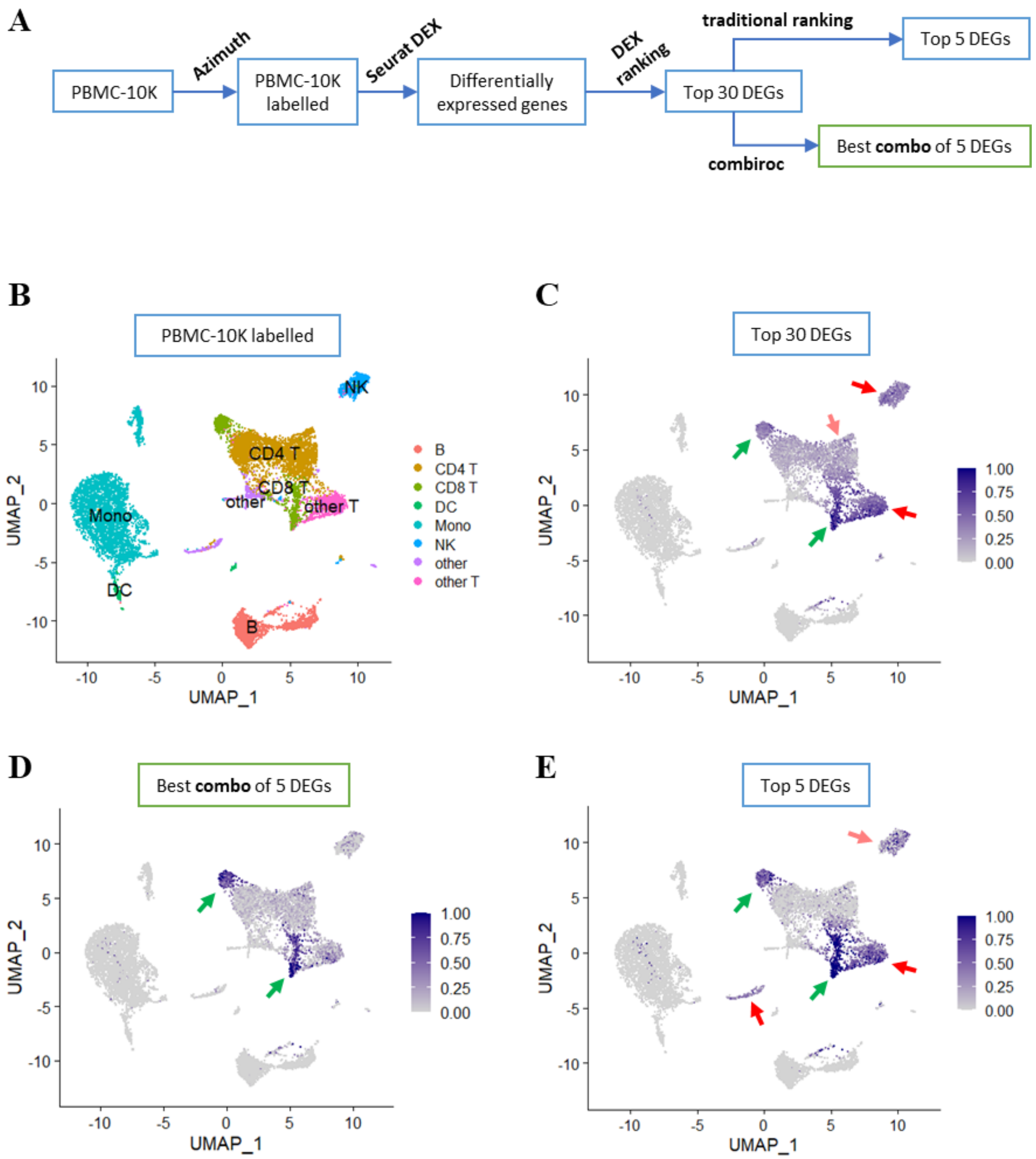

Fig. S4. CD8-T cells gene signature refinement on PBMC-10K dataset. (**A**) general workflow of cluster classification (Azimuth), differential expression (DEX with Seurat v.4), gene signature selection by fold change and refinement (combiroc). (**B**) UMAP plot of PBMC-10K cells with cell types annotated by Azimuth. CD8-T cells are in green. (**C**) Cumulative expression of the 30 most differentially expressed genes in CD8-T cells. (**D**) Cumulative expression of the 5 genes in the combiroc combination (CD8A, CD8B, GZMH, LINC02446, TRAC). (**E**) Cumulative expression of the 5 most differentially expressed genes in CD8-T cells (CD8A, CD8B, GZMH, CCL5, CD3D). Red

arrows show marker genes expression falling outside the CD8-T cell cluster (false positives), green arrows indicate markers expression correctly positioned on CD8-T cell cluster (true positives).
